# Cashew Gum Polysaccharide Nanoparticles Grafted with Polypropylene Glycol as Carriers for Diclofenac Sodium

**DOI:** 10.3390/ma14092115

**Published:** 2021-04-22

**Authors:** Cassio Nazareno Silva da Silva, Maria Carolina Bezerra Di-Medeiros, Luciano Morais Lião, Kátia Flávia Fernandes, Karla de Aleluia Batista

**Affiliations:** 1 Laboratório de Química de Polímeros, Instituto de Ciências Biológicas, ICB2, Campus Samambaia, Universidade Federal de Goiás, Goiânia 74690-900, GO, Brazil; cassio1994@hotmail.com (C.N.S.d.S.); kfernandes.lqp@gmail.com (K.F.F.); 2 Laboratório de Ressonância Nuclear Magnética, Universidade Federal de São Carlos, São Carlos 13565-905, SP, Brazil; caroldimedeiros@gmail.com; 3 Laboratório de Ressonância Nuclear Magnética, Campus Samambaia, Universidade Federal de Goiás, Goiânia 74690-900, GO, Brazil; luciano@quimica.ufg.br; 4 Departamento de Áreas Acadêmicas, Instituto Federal de Educação, Ciência e Tecnologia de Goiás, Campus Goiânia Oeste, Goiânia 74270-040, GO, Brazil

**Keywords:** CGP, polymeric nanoparticles, release, drug delivery, encapsulation, NSAID

## Abstract

This investigation focuses on the development and optimization of cashew gum polysaccharide (CGP) nanoparticles grafted with polypropylene glycol (PPG) as carriers for diclofenac sodium. The optimization of parameters affecting nanoparticles formulation was performed using a central composite rotatable design (CCRD). It was demonstrated that the best formulation was achieved when 10 mg of CGP was mixed with 10 μL of PPG and homogenized at 22,000 rpm for 15 min. The physicochemical characterization evidenced that diclofenac was efficiently entrapped, as increases in the thermal stability of the drug were observed. The CGP-PPG@diclofenac nanoparticles showed a globular shape, with smooth surfaces, a hydrodynamic diameter around 275 nm, a polydispersity index (PDI) of 0.342, and a zeta potential of −5.98 mV. The kinetic studies evidenced that diclofenac release followed an anomalous transport mechanism, with a sustained release up to 68 h. These results indicated that CGP-PPG nanoparticles are an effective material for the loading/release of drugs with similar structures to diclofenac sodium.

## 1. Introduction

The use of nanoparticulated drug delivery systems has gained interest in the last decades due to the possibility of control the rate and period of drug delivery [1,2]. These nanoparticulated materials has as an advantage the increase of bioavailability, solubility, and permeability of different drugs molecules that are otherwise difficult to deliver orally [3,4,5]. Indeed, the development of polymeric nanoparticles containing therapeutic agents has attracted considerable interest in scientific and commercial fields due to their capability to efficiently control drug release and improve the specificity with the target site, while protecting therapeutic molecules from degradation activities. In addition, the nanoparticulated delivery systems are more efficient in avoiding undesired side effects (i.e., allergic reactions, stomach ulcers, kidney and liver problems), when compared to conventional formulation [4,5,6]. In addition, the possibility of preparation of polymeric nanoparticles using the self-assembly methodology is quite interesting since it comprises a cost-effective and versatile process for the production of stable and robust structures [7,8].

In this context, cashew gum polysaccharide (CGP) stands out as a promising polymer to be used as a drug nanocarrier due to its nontoxicity, biocompatibility, and biodegradability, representing a self-renewable biobased source [9,10]. CGP is a complex acid polysaccharide isolated from the exudate of cashew threes, being composed of a main chain of β-galactose (1⟶3), with branches of β-galactose (1⟶6). Its main constituents are glucose, rhamnose, arabinose, and glucuronic acids [11,12].

Copolymers of CGP have been studied for several purposes, especially in the preparation of different delivery vehicles for hydrophobic and hydrophilic active molecules [13,14,15]. In this paper, we report for the first time the use of self-assembled nanoparticles of CGP grafted with polypropylene glycol (PPG) for controlled drug release. Polypropylene glycol (PPG) is a polymer frequently used in the production of biomedical devices, being less toxic and cheaper than polyethylene glycol (PEG). In addition, PPG presents high chemical stability; it shows interesting mucoadhesive features and is a safe, biodegradable, and biocompatible polymer. Small amounts of PPG do not affect human health, since the product of PPG catabolism is pyruvic acid, which is a common metabolite used to produce metabolic energy [3,16]. These self-assembled nanoparticles were designed to entrap diclofenac sodium aiming to provide an oral delivery system, and they have the potential to reduce the undesired side effects associated with the conventional administration of this drug, such as gastrointestinal upset, which increases the risks of gastric and duodenal ulcer as well as hemorrhage [13,14].

Diclofenac sodium is a potent nonsteroidal anti-inflammatory drug (NSAID) widely used as an analgesic and antipyretic drug for the treatment of acute pain and chronic inflammatory processes [15]. Although diclofenac is considered a potent anti-inflammatory drug, it presents important drawbacks, especially regarding its short biological half-life, its high proportion of protein binding, and its highly presystemic metabolism [16]. According to the Biopharmaceutical Classification System, diclofenac shows limited solubility in water and aqueous fluids, being classified in Group II of therapeutics. In addition, diclofenac absorption is dependent of its dissolution rate and solubility, properties which will dictate its bioavailability [17,18]. Due to the short biological half-life and associated undesired side effects, diclofenac is an interesting candidate to be used in the development of controlled drug delivery systems for improving its therapeutic efficacy and patient compliance [15,19,20]. In the last years, diverse delivery systems have been proposed to increase the efficacy of diclofenac, with a number of studies focusing on the development of polymeric membranes and films [21,22], polymeric micro/nanoparticles [23], and hydrogels [24,25].

In this work, the production of self-assembled nanoparticles of CGP grafted with polypropylene glycol (PPG) for the entrapment of diclofenac sodium, as well as the release of this drug, was investigated. A central composite rotatable design (CCRD) coupled with response surface methodology (RSM) was used to model, evaluate, and optimize the polymer concentrations and process parameters involved in the formulation of nanoparticles containing diclofenac sodium as a model drug. The prepared CGP-PPG@diclofenac nanoparticles were characterized and the drug release behavior was evaluated as a proof-of-concept for a drug delivery system.

## 2. Materials and Methods

### 2.1. Chemical and Reagents 

Polypropylene glycol (molecular weight ~2000) and diclofenac sodium salt was purchased from Sigma-Aldrich Chemical Co. (St. Louis, MO, USA). All other chemicals used were of analytical grade, obtained from accredited companies and were used as received.

### 2.2. Cashew Gum Polysaccharide Isolation

Exudates from cashew tree (*Anacardium occidentale*) were collected in July 2018 in the city of Aquirás, Ceará, Brazil. The cashew gum was milled, dispersed in distilled water in a proportion of 1:5 (*w*/*v*) and kept at room temperature (25 °C), for 24 h, under continuous stirring. The dispersion was filtered in nylon membranes to remove impurities and the solubilized polysaccharide was precipitated with cold ethanol in the proportion of 1:3 (*v*/*v*). The precipitated polysaccharide (CGP) was isolated by centrifugation, washed with cold ethanol and dried at room temperature (25 °C). The dried polysaccharide was milled and stored in airtight vials.

### 2.3. Nanoparticles Preparation

In this study, a 2^3^ central composite rotatable design (CCRD) coupled with response surface methodology (RSM) was employed to model, optimize, and evaluate the effects and interactions of the polymer’s content and rotational speed on the nanoparticle hydrodynamic diameter and encapsulation efficiency of diclofenac sodium. The parameters and the levels were defined as described in Table 1. A central point with two replicates was also added for statistical evaluation. 

Results from CCRD were analyzed using the software Statistica 10.0 (Statsoft Inc., Tulsa, OK, USA, 1997). Experimental data were submitted to regression analysis coupled with response surface methodology, and the adjustment of the data in the RSM was represented by the following second-order polynomial equation (Equation (1)):

(1) $$\;y=\beta_{0}+\sum_{j}\beta_{j}x_{j}+\sum_{i<j}\beta_{ij}x_{i}x_{i}+\sum_{j}\beta_{jj}x^{2}+e,$$

where y is the response (dependent variable) to be modeled;$\;\beta_{0},\;\;\beta_{j}$, $\beta_{ij}$, and $\beta_{jj}$ are regression coefficients, $x_{j}$ and $x_{i}$ are parameters used in the nanoparticles production (independent variables), and *e* is the error. After ANOVA analysis, the model was simplified by dropping terms that were not statistically significant (*p* = 0.05). Desirability function was performed, and the optimum formulation was based on set criteria of maximum entrapment efficiency (EE) with minimum hydrodynamic diameter. 

For the preparation of the nanoparticles, diclofenac sodium and PPG were dissolved in 1 mL of ethanol and slowly dropped to 10 mL of an aqueous solution of CGP. The mixture was homogenized for 15 min using an Ultra-Turrax^®^ T25 homogenizer (Ika^®^-Werke GmbH & Co.KG, Staufen im Breisgau, Germany), equipped with the S25N-18G dispenser tool.

### 2.4. Entrapment Efficiency

The encapsulation efficiency of the nanoparticles was measured by recording the drug concentration in the supernatant solution using ultraviolet spectrophotometry. All measurements were made at least in triplicate by the use of four separate specimens. Samples of 50 μL were taken and after dilution to 5 mL with distilled water, their absorbance was measured with a spectrophotometer (BEL photonics 2000) (BEL Engineering, Piracicaba, SP, Brazil), at 280 nm, and compared to a calibration curve in purified water (linear range: 10–70 mg/L; r^2^ = 0.9953). The encapsulation efficiency (EE) was calculated according to Equation (2):

(2) $$EE\;\left(\%\right)=\frac{W_{i}-W_{f}}{W_{i}}\times 100,$$

where $W_{i}$ represents the amount of diclofenac sodium used in the production of CGP-PPG nanoparticles, and $W_{f}$ is the final amount of free diclofenac sodium in the supernatant.

### 2.5. Hydrodynamic Diameter and Zeta Potential

The hydrodynamic diameter and polydispersity index (PDI) were investigated by dynamic light scattering in the backscatter mode with a ZetaSizer Nano (ZEN 1600, Malvern Instruments Ltd., Malvern, UK). For the determination of the hydrodynamic diameter, an aliquot of 10 μL was diluted to 1 mL before analysis. The dispersant used was water at 25 °C. The system was calibrated before each measurement, and the measurements were performed using a fixed dispersion angle of 173°. The ζ-potential was determined with the same device by laser doppler microelectrophoresis in the forward scattering mode. For the analysis of ζ-potential, the samples were kept in pH 7.0. All measurements were accomplished in triplicate.

### 2.6. Scanning Electron Microscopy (SEM)

The structure of CGP-PPG@diclofenac nanoparticles was analyzed by scanning electron microscopy (SEM), using a secondary electron detector with an accelerating voltage of 8 kV (JEOL JSM-IT300) (Jeol Ltd., Welwyn Garden City, Hertfordshire, UK). A drop of CGP-PPG@diclofenac solution was dropped onto a glass slide and dried in a desiccator (Labsynth, São Paulo, SP, Brazil). After drying, the slide was sputter-coated with a layer of gold under vacuum. The particle size was determined using the Fiji software [26]. The average particle size was determined after at least 20 individual measures by open field, with five different fields evaluated during SEM analysis. SEM experiments and analyses were performed in the Laboratório Multiusuário de Microscopia de Alta Resolução (LabMic) at the Universidade Federal de Goiás, GO, Brazil. 

### 2.7. Fourier-Transform Infrared Spectroscopy (FTIR)

FTIR spectra of polymers, diclofenac, and nanoparticles were acquired on a PerkinElmer FTIR spectrometer (Spectrum 400, PerkinElmer, Inc., Waltham, MA, USA). For the test, samples were mixed with potassium bromide (KBr), and the pellets were analyzed in the range of 4000–400 cm^−1^ (resolution of 2 cm^−1^), with 12 scans recorded. 

### 2.8. X-ray Diffraction (XRD) Analysis

X-ray diffraction measurements of the samples were performed using XRD diffractometer (D8 Discover, Bruker AXS, Billerica, MA, USA). One-dimensional, wide-angle diffraction patterns were obtained using a radiation source of Cu (Kα_1_ = 1.54 Å), at a voltage of 40 kV and a current of 40 mA. The samples were scanned at the diffraction angle (2θ) from 5° to 80° (scanning rate = 0.01°). All samples were dried in vacuum and then stored at room temperature (25 °C) before measurements. 

### 2.9. In Vitro Drug Release Studies

The in vitro release tests were performed according the methodology described by Cheikh et al. [19], using Franz diffusion cells (Spell, Minas Gerais, Brazil) presenting 13.4 mm in diameter. Dialysis membranes with a molecular cutoff value of 14 kDa (Sigma-Aldrich Chemical Co., St. Louis, MO, USA) were positioned between upper and lower chambers. Five milliliters of a solution of CGP-PPG@diclofenac nanoparticles (containing 3.5 mg of diclofenac sodium entrapped) were applied to the upper donor chamber. In the lower receiver chamber, we added a phosphate buffered saline (PBS, pH 6.8) that was continuously stirred at 300 rpm. The joint between the upper and lower chambers was clamped tightly to avoid fluid loss. The temperature of the Franz diffusion cells was maintained using a water circulator (Refrigerated Bath Circulator, model SD07R-20, PolyScience, Niles, IL, USA), at 37 ± 2 °C. Aliquots of 1 mL were collected in the receiver chamber at regular time intervals up to 68 h, and after each sampling the same volume of fresh PBS was added to maintain the total volume in the receiver chamber. The diclofenac concentration was determined by measuring the absorbance with a spectrophotometer (BEL photonics 2000, BEL Engineering, Piracicaba, SP, Brazil), at 280 nm, and we compared the readings to a calibration curve in PBS pH 7.4 (linear range: 5–100 mg/L; r^2^ = 0.993). The release of diclofenac sodium was calculated as a percentage.

### 2.10. Drug Release Kinetics

To analyze the mechanism of drug release, several models were applied to adjust the experimental data, using KinetDS 3.0 software [27]. The release pattern of drug sodium from the CGP-PPG@diclofenac nanoparticles was determined by fitting the release profile to zero-order, first-order, Higuchi and Korsmeyer–Peppas models. The zero-order model is expressed as the cumulative amount of drug released versus time, as determined by Equation (3):

(3) $$C=k_{0}t,$$

where *t* represents time (min) and *k*_0_ represents the zero-order rate constant, obtained from the slope of the graphical representation of concentration vs. time.

The first-order model can be defined as the logarithm plot of the cumulative percentage of drug remaining as a function of time. This model can be expressed by Equation (4):

(4) $$logC=logC_{0}-k\cdot \left(t/2.303\right),$$

where *C*_0_ is the initial drug concentration, *k* is the first order constant, and *t* is the time (min).

Higuchi’s model can be defined as the cumulative percentage of drug released versus the square root of time, according to Equation (5):

(5) $$Q_{t}=k\cdot t^{1/2},$$

where *Q_t_* represents the content of drug released in time *t*, *k* represents the kinetic constant, and *t* represents the time (min).

The Korsmeyer–Peppas model was also used to discriminate the different mechanisms of drug release. The data from diclofenac release were fitted to the Peppas exponential model, and the release exponent *n* and *k* value were determined using Equation (6):

(6) $$M_{t}/M_{\infty }=k\cdot t^{n},$$

where *M_t_* refers to the amount of the drug released at time *t*, *M_∞_* represents the total of drug released after an infinite time, *k* corresponds to the diffusional characteristic of the drug/nanopolymer system constant, and *n* represents the exponent describing the diffusional mechanism. In general, values of *n* ≤ 0.5 indicate that the release mechanism follows a Fickian diffusion. For 0.5 < *n* ≤ 1.0, the releasing mechanism follows a non-Fickian model (anomalous transport). When *n* > 1.0, the drug release follows a case II transport mechanism [28].

## 3. Results and Discussion

### 3.1. Production of CGP-PPG@Diclofenac Nanoparticles

The use of CCRD coupled with RSM is considered as the alternative approach for optimizing the production of nanoparticles since it is possible to analyze several variables at different levels with a reduce number of experiments [29,30]. Herein, sixteen batches were formulated according to CCRD-RSM, and the results of the hydrodynamic diameter, polydispersity index, and zeta potential of the particles and drug entrapment efficiency are shown in Table 2. As can be observed, the produced particles presented a diameter varying from 544 nm to 10.08 μm, with values of PDI ranging from 0.43 to 1, and a zeta potential from −0.08 to −25.3 mV.

**Figure 1:**
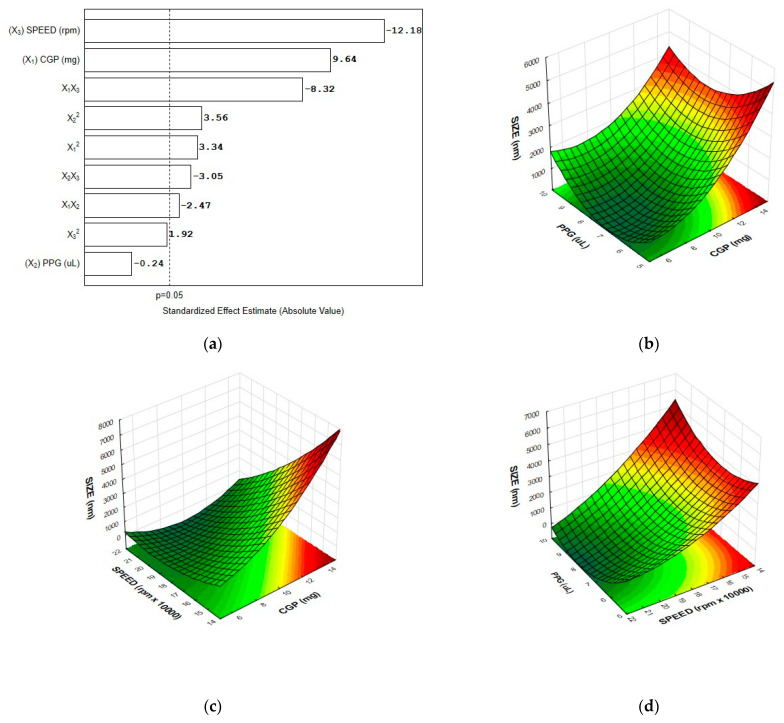
Pareto chart (**a**) and response surface plot for hydrodynamic diameter (size) as a function of (**b**) CGP content and PPG volume; (**c**) CGP content and homogenization speed; and (**d**) PPG volume and homogenization speed (*n* = 6 replicates).

Results of the multivariate analysis evidenced that the polymers content and the speed of homogenization affected the hydrodynamic diameter of the particles (*p* < 0.05). As can be seen in the Pareto chart (Figure 1a), the linear (X_1_) and quadratic (X_1_^2^) terms for CGP content and the quadratic term for PPG volume (X_2_^2^) caused a significative increase in the particle diameter. On the other hand, the linear effect for the speed of homogenization (X_3_) and the interaction effects between the polymers (X_1_X_2_) and between polymers and speed (X_1_X_3_ and X_2_X_3_) provoked a reduction in the hydrodynamic diameter of the CGP-PPG@nanoparticles. 

The regression analysis shows an adequate fit of experimental values to the second-order polynomial model as a function of significant factors (r^2^ = 0.91; adjusted r^2^ = 0.90). The mathematical model is represented in Equation (7):

(7) $$\mathrm{Hydrodynamic}\;\mathrm{diameter}\;(\mathrm{nm})=1230.X_{1}^{}+47.4X_{1}^{2}\;+202.1X_{2}^{2}-40.3X_{1}X_{2}-84.7X_{1}X_{3}-62.1X_{2}X_{3},$$

where $X_{1}$ denotes the content of CGP (mg), $X_{2}$ denotes the volume (μL) of PPG, and $X_{3}$ denotes the speed (rmp × 1000) of homogenization. 

In order to understand the interrelation and interaction effects between the factors affecting the hydrodynamic diameter of the CGP-PPG@diclofenac nanoparticles, three-dimensional plots were created using the results of the regression model, in which one factor was fixed at the center level, and the other two variables were varied within the experimental range. As can be observed in Figure 1b, the hydrodynamic diameter of the particles was strongly affected by the CGP content. The branched structure and the hydrophilic character of CGP spatially expands the particle chemical interaction with the aqueous surrounding environment, therefore increasing the hydrodynamic diameter of the CGP-PPG@diclofenac particles. In addition, the hydrodynamic diameter reduces as the homogenization speed increases (Figure 1c,d), which was expected due to the increasing shearing [31]. During the mechanical stirring using the Ultra-Turrax homogenizer, the high accelerations acting in the polymer molecules produces extremely strong shear and thrust forces, highly increasing the turbulence between the rotor and stator (shear gap), which leads to a size reduction of the produced nanoparticle. Similar results were obtained for Bhadra et al. [32], Burapapadh et al. [33], and Zhang et al. [34], who evidenced the effectiveness of the use of shear-based homogenization to reduce the size of nanoparticles with different compositions.

### 3.2. Entrapment Efficiency

In order to evaluate the feasibility of CGP-PPG nanoparticles to act as a diclofenac sodium carrier, the effect of formulation variables and process parameters such as CGP content, PPG volume, and homogenization speed on entrapment efficiency were investigated using CCRD, and the results are shown in Table 2. The efficiency of diclofenac retention indicates the amount of diclofenac entrapped and retained in the nanoparticle system. As can be observed, the nanoparticle composition affected the drug entrapment differently. However, the entrapment efficiency was higher than 70% for all tested formulations, which evidences that CGP-PPG nano- and microparticles are promising as drug carriers. 

The data from entrapment efficiency (EE) were fitted to the second-order polynomial model, and the correlations between the response and the variables were expressed by Equation (8):

(8) $$\mathrm{Entrapment}\;\mathrm{efficiency}\;(\%)=145.4+4.10X_{1}^{}-0.27X_{1}^{2}-11.90X_{2}^{}+0.40X_{2}^{2}-3.1X_{3}^{}+0.06X_{3}^{2}+0.02X_{1}X_{2}+0.08X_{2}X_{3},$$

where $X_{1}$, $X_{2}$, and $X_{3}$ denotes the content of CGP (mg), the volume of PPG (μL), and the speed of homogenization (rpm × 1000), respectively. The value of r^2^ (0.967) indicates that the variability of the response is effectively explained by the experimental model. In addition, the value for the adjusted r^2^ (0.96) confirms the fitness of the model, indicating that 96% of the experimental data were in agreement with the predicted values. 

**Figure 2:**
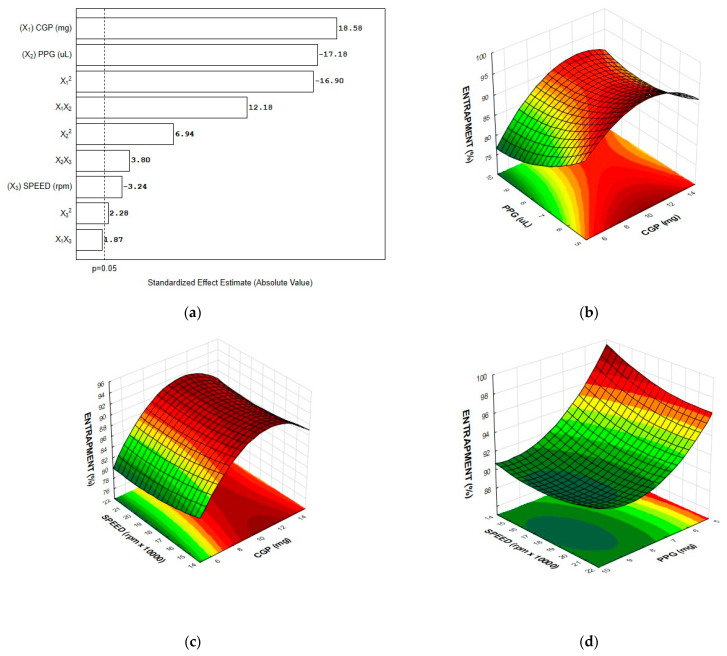
Pareto chart (**a**) and response surface plot for diclofenac entrapment efficiency as a function of (**b**) CGP content and PPG volume; (**c**) CGP content and homogenization speed; and (**d**) PPG volume and homogenization speed (*n* = 6 replicates).

Results from the multivariate analysis evidenced that all variables interfered with the entrapment efficiency of diclofenac sodium. The evaluation of the relationship between the dependent and independent variables shows that the most pronounced effect was observed for the linear term of CGP content, positively affecting the response (Figure 2a). In addition, the quadratic terms for PPG volume (X_2_^2^) and homogenization speed (X_3_^2^) and the interaction factors X_1_X_2_ and X_2_X_3_ also had a positive effect on the response, contributing to increase the content of diclofenac entrapped in the CGP-PPG@nanoparticle. On the other hand, the linear terms for PPG volume (X_2_) and homogenization speed (X_3_) and the quadratic term for the CGP content (X_1_) negatively affected the entrapment efficiency. 

The three-dimensional plots showing the individual effects of polymers’ content and homogenization speed as well as their interactions on the entrapment efficiency of diclofenac sodium are shown in Figure 2. The response surfaces plots depicted in Figure 2b,c show an overall curvilinear response, with a plateau of maximum entrapment efficiency for the nanoparticles presenting around 10 mg of GCP. Drug entrapment efficiency strongly depends on the solid-state drug solubility in the polymer matrix, which is linked to the composition of polymer, the molecular weight, the drug–polymer interaction, and the presence of end functional groups. In this case, it is possible that dipole–dipole interactions between the diclofenac and CGP molecules might favor drug loading. This attraction between the oppositely directed dipoles from CGP (δ+: hydrogen from hydroxyl groups) and diclofenac (δ−: chlorine and carboxylic oxygen) can cause changes in the internal structure of the polymeric nanoparticle with diclofenac penetrating deeply into the polymeric chain of CGP [35]. On the other hand, the higher the PPG content, the lower the percentage of drug entrapment (Figure 2a,d). This effect is most probably related to the self-assembling process rather than the PPG’s inherent characteristic. It is also possible that PPG acts in a way causing steric hindrance that reduces the attractive forces between diclofenac sodium and CGP, then reducing the available sites for diclofenac sodium interaction and hence its entrapment. 

**Figure 3:**
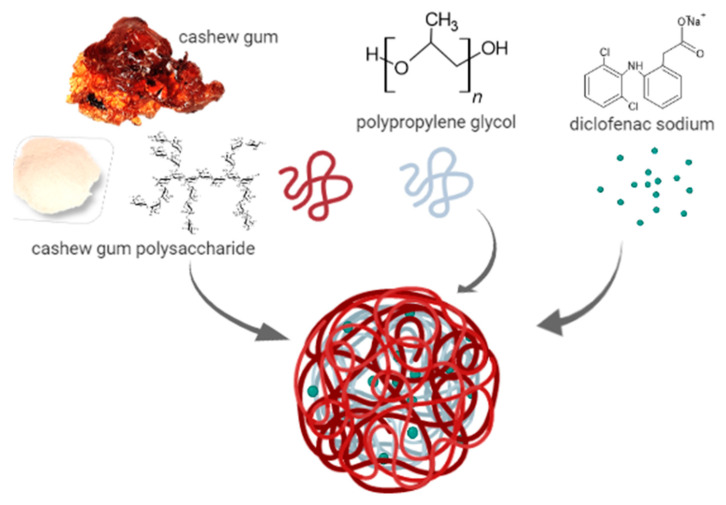
Schematic representation of the polymers and drug organization in the CGP-PPG@diclofenac nanoparticles.

In addition, the curvatures observed in the response surface plots suggest that there are interactions between the factors (Figure 2b–d). The existence of interaction reveals that the factors may influence the diclofenac entrapment interactively and not independently. It is possible that during the self-assembling of the polymers, the diclofenac molecules are entrapped between the side chains of CGP molecules, and that this entrapment is dependent of the available sites of interaction (Figure 3). Considering the hydrophobic characteristic of PPG, it competes by the inner hydrophobic spots in the CGP molecules, negatively interfering with the diclofenac entrapment. This can explain the best results of entrapment observed at the lowest content of PPG (Figure 2b) used for the production of the CGP-PPG@diclofenac nanoparticles. Additionally, increases in the homogenization speed will lead to the production of particles with increased area for entrapment, which results in a higher diclofenac loading in the nanoparticles (Figure 2c,d).

### 3.3. Optimization of the Nanoparticle Formulation and Model Validation

The nanoparticle size is a key factor during the optimization procedures, since it is expected interfere with the biopharmaceutic characteristics of the produced material, in turn influencing the release profile of the encapsulated biomolecule. In general, particles higher than 4000 nm can cause capillary occlusion and embolism. Additionally, particles with a higher size are easily recognized by the mononuclear phagocyte system, as they are removed from the body before they can perform their designed therapeutic function [36]. In this sense, it is desirable that nanoparticles aimed to be used as drug delivery systems present a size lower than 600 nm [37,38]. 

In order to achieve a nanoparticle formulation presenting the lowest hydrodynamic diameter, optimization by the desirability function method was used. Desirability is an optimization method used to group the effect of all variables aiming to provide an objective function representing the relationship of all responses obtained during optimization [39]. The mathematical prediction for the highest desirability suggested as production parameters: 10 mg of CGP and 10 μL of PPG, with a homogenization speed of 22,000 rpm (d = 0.95). At these conditions, it was expected that particles have a hydrodynamic diameter around 300 nm. The model was experimentally validated to confirm the mathematical prediction, and the produced nanoparticles presented a diameter around 275 ± 15.9 nm, which was better than that calculated by the desirability model. In addition, the polydispersity index (PDI), zeta potential, and entrapment efficiency for the optimized formulation were evaluated. Results evidenced that the CGP-PPG@diclofenac nanoparticles showed a PDI around 0.342 (±0.01), suggesting that the system is moderately polydisperse [40,41]. The surface charge of the NPs was negative with a ζ-potential of −5.98 ± 0.79 mV, and an entrapment efficiency of 95.6% was achieved.

### 3.4. Nanoparticles Characterization

#### 3.4.1. Scanning Electron Microscopy (SEM)

**Figure 4:**
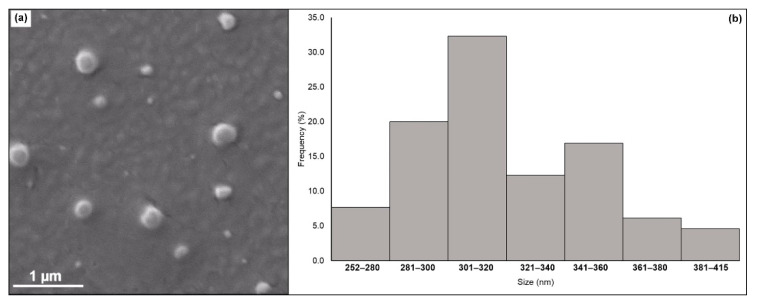
(**a**) SEM micrograph and (**b**) particle size distribution of the CGP-PPG@diclofenac nanoparticles.

The morphology of the CGP-PPG@diclofenac nanoparticles was evaluated by SEM (Figure 4). The nanoparticles were in general globular with smooth surfaces. There was no particle aggregation (Figure 4a), and the particle sizes determined by SEM varied from 252 to 412 nm (Figure 4b), with an average size of 321 ± 33 nm, a value close to that predicted by the desirability function. The nanoparticle morphology is considered a crucial element in the development and design of commercial nanoparticles, which can influence in vivo pharmacokinetics and cell uptake and consequently might interfere with the biological application efficacy [2,42,43]. The lack of roughness in polymeric nanoparticles is generally associated with the elastic nature of the polymer, which adapts to different shapes, thus reducing possible surface imperfections [44].

#### 3.4.2. FTIR Spectroscopy Analysis

**Figure 5:**
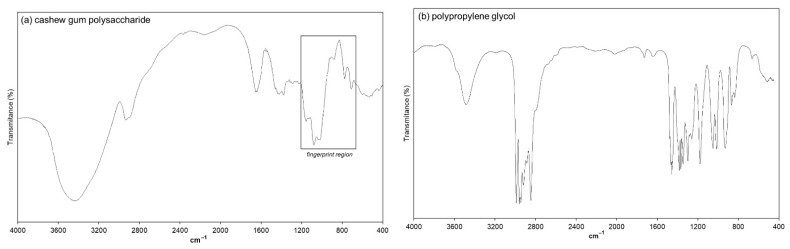

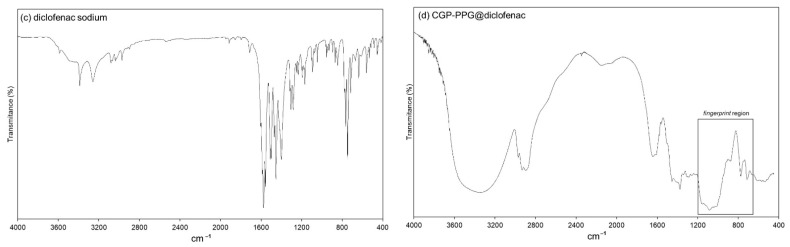
FTIR spectra of (**a**) cashew gum polysaccharide (CGP), (**b**) polypropylene glycol (PPG), (**c**) diclofenac sodium, and (**d**) CGP-PPG@diclofenac nanoparticles.

The FTIR spectral data of pure CGP, PPG, and diclofenac are shown in Figure 5. The FTIR spectrum of CGP showed the characteristic peaks associated with this polysaccharide. As can be seen in Figure 5a, the broad band around 3438 cm^−1^ is characteristic of the stretching vibration of hydroxyl groups, and the band at 2936 cm^−1^ is relative to C–H stretching [45]. The peak at the 1421 cm^−1^ frequency is related to the symmetric stretching of carboxylic groups (–COO^−^) in the polysaccharide structure [12,46,47]. In addition, the frequencies around 1100–700 cm^−1^ comprises the “fingerprint region” commonly associated with the polysaccharide structure. The peaks at approximately 1080 and 710cm^−1^ are related to the glycosidic linkage (C–O–C) vibrations and the stretching vibrations of –OH of the polysaccharide. It is also possible to observe a peak around 1640 cm^−1^ due to O–H scissor vibrations from bound water molecules [47].

The FTIR spectrum of pure PPG (Figure 5b) evidences a peak around 3480 cm^−1^ attributed to the stretching vibration from the terminal –OH groups of the PPG. Characteristic intense peaks around 2992–2841 cm^−1^ are related to the symmetric and asymmetric axial deformations of –CH_3_ groups and the axial symmetric deformation of –CH_2_ groups [48]. The bands at approximately 1175 and 866 cm^−1^ are related to the asymmetric and symmetric stretching of C–O–C, respectively [46,49]. 

The FTIR spectrum of pure diclofenac sodium is presented in Figure 5c. It is possible to observe a band at 3387 cm^−1^ related to –NH stretching of the secondary amine. The characteristic peaks at 1574 cm^−1^ and 1398 cm^−1^ are assigned to the asymmetric and symmetric carboxylate stretching vibration in the diclofenac structure, respectively. A peak at 1282 cm^−1^ due to the C–N stretching vibration of aromatic amine was also observed, as well as a peak at 746 cm^−1^ related to the carbon and chloride stretching vibration of chlorinated benzene [46,50,51]. 

The interaction between the drug and polymers is commonly identifiable by changes in the FTIR profile. The FTIR spectrum of CGP-PPG@diclofenac nanoparticles is demonstrated in Figure 5d. The interaction at a molecular level between CGP and PPG chains could be evidenced by the changes in the peaks around 3438 cm^−1^ in CGP and 3480 cm^−1^ in PPG. In the spectrum of CGP-PPG@diclofenac nanoparticles, this band becomes wider and shifts to a lower wavenumber (3363 cm^−1^), which might indicate that besides the presence of a different content of entrapped water, there is also an enhancement of the hydrogen bond interactions [52]. In addition, the fingerprint region (1100–700 cm^−1^) assigned to the CGP structure was maintained, while the characteristic peaks related to the PPG structure (1053–866 cm^−1^) disappeared, indicating that PPG molecules must be located in the inner part of the nanoparticle, protected from the contact of an aqueous environment. 

It is possible that the backbone of the CGP molecule is interacting with PPG and diclofenac while its side chains are organized like bristles of CGP extending from the surface, conferring a brush-like conformation to the nanoparticle [52,53,54]. Nanoparticles presenting a brush-like conformation generally present a prolonged retention time in circulation. The arrangement of the CGP ramifications into brush configuration generates greater protein repulsion, causing CGP to avoid interactions between the nanoparticles and plasma proteins, which enhances the lifetime of the molecule in circulation by reducing the rate of blood clearance of this nanoparticle [3,54]. Furthermore, the carbon nitrogen stretching vibration peak of aromatic amine (1282 cm^−1^) and the carbon chlorine stretching vibration peak of chlorobenzene (746 cm^−1^) from diclofenac sodium were not observed in the CGP-PPG@diclofenac nanoparticle’s spectrum (Figure 5b), indicating that the entrapment of the drug occurred on the inner structure of the nanoparticle (Figure 3).

#### 3.4.3. XRD Analysis

**Figure 6:**
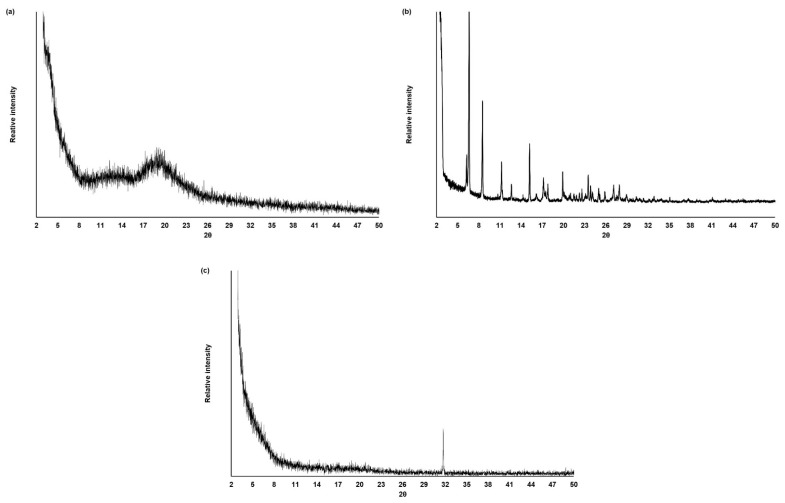
XRD diffractograms of the (**a**) cashew gum polysaccharide (CGP), (**b**) diclofenac sodium, and (**c**) CGP-PPG@diclofenac nanoparticles.

X-ray diffraction (XRD) is frequently used to analyze the degree of sample crystallinity. As indicated by XRD results, CGP and diclofenac presented quite a different crystallinity degree. As expected, the gummous nature of pure CGP is characterized by the presence of a broad reflection at 2θ = 18.8° in the diffractogram (Figure 6a), confirming the amorphous nature of this polysaccharide [45]. On the opposite, the pure diclofenac sodium diffractogram (Figure 6b) presented distinct 2θ diffraction peaks at 6.5, 8.5, 11.2, 12.5, 15.3, 17.1, 17.8, 19.9, 23.5, 27.1, and 27.9. These peaks coincide with the salt form of diclofenac, evidencing its crystalline structure [55,56].

CGP-PPG@diclofenac nanoparticles were characterized by the absence of the diffraction peaks from diclofenac, with a single diffraction peak observed at 2θ = 31.9°. This absence of characteristic reflections from diclofenac might indicate a drug amorphization or its solvation in the amorphous carrier (Figure 6c). The X-ray diffractograms corroborate the finding obtained from the FTIR analysis (Figure 5), indicating that diclofenac sodium is entrapped in the inner structure of the produced nanoparticles (Figure 3).

### 3.5. In Vitro Drug Release Study

**Figure 7:**
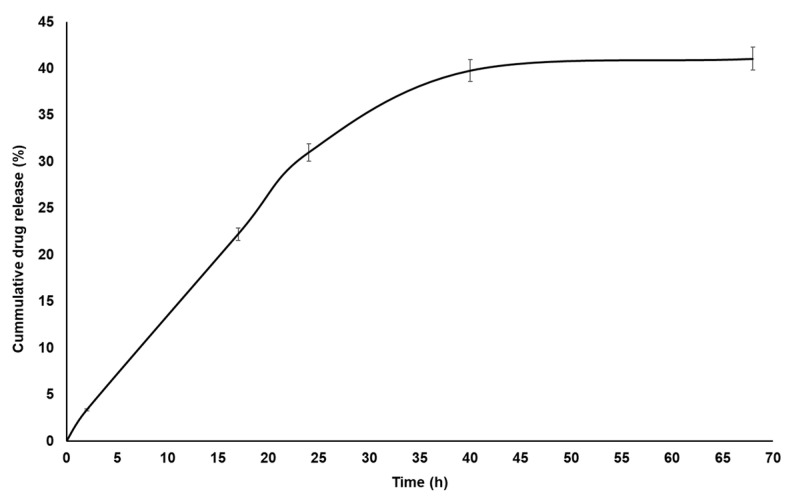
In vitro drug release profile of the diclofenac sodium loaded into CGP-PPG@diclofenac nanoparticles (*n* = 6 determinations).

In order to evaluate the potential of the produced nanoparticles for controlled drug release, the CGP-PPG@diclofenac nanoparticles were evaluated regarding the release profile of diclofenac sodium over 68 h. The experiment was performed in simulated intestinal conditions (pH 6.8) since it is the common site of the absorption of drugs orally administered. As can be seen in Figure 7, a slow and gradual release of diclofenac sodium occurred until 50 h, with a cumulative release of 41% observed. After this point, a plateau of sustained release until 68 h was observed. This controlled and prolonged release profile of diclofenac from the CGP-PPG@diclofenac nanoparticles could be interesting to explore for avoiding the “peak and valley” problems commonly associated with the conventional oral administration of this drug, and this would contribute to increasing the efficiency of the therapy while reducing the undesired side effects associated with the use of diclofenac sodium.

Similar release profiles were also observed by using other diclofenac release systems. For instance, Dias et al. [57] reported a slow release rate of diclofenac diethylamine encapsulated in acetylated cashew gum nanoparticles. Despite the higher accumulated release reported by these authors (60%), the release profile observed in our work was more homogeneous, without the presence of burst effect and able to maintain a gradual and sustained release for more than 24 h. Compared to other polymeric nanostructured delivery systems for diclofenac, the CGP-PPG@diclofenac presented a performance similar to those from Yahia et al. [16] and Duarte Junior et al. [20], thus confirming the capacity of chitosan particles loaded with diclofenac sodium to provide a sustained release of this drug. Similarly, Yousefi et al. [58] showed that magnetic@layered double hydroxide multicore@shell nanostructures were effective as controlled delivery systems for diclofenac and ibuprofen.

The mechanism through which diclofenac was released from the nanoparticles was analyzed with mathematical models of zero-order, first-order, Higuchi and Korsmeyer–Peppas (Table 3). From these results, the diclofenac release was better adjusted to the Korsmeyer–Peppas model, which presented the lowest AIC (Akaike information criterion) and the highest r^2^ (coefficient of determination). Similar release mechanism were found for kaolinite/CGP nanoparticles containing antihypertensive molecules [45], acetylated CGP nanoparticles containing diclofenac diethylamine [57], and indomethacin [59].

After applying the data from the curve of Figure 7 to the Korsmeyer–Peppas equation, the release exponent (*n*) value was obtained. The “*n*” exponent is used to suggest the mechanism of drug release, and for materials with a spherical form, values of *n* ≤ 0.43 indicate a release dictated by a Fickian diffusion; for 0.43 < *n* < 0.85, the release is related to an anomalous transport; and when *n* ≥ 0.85, the drug release is related to a polymer swelling or case II transport [59,60,61]. An “*n*” exponent value of 0.84 was found for diclofenac sodium, indicating that drug release occurred according to an anomalous transport, in which this release process is driven by both swelling of the CGP-PPG nanoparticle and diffusion of the drug. Furthermore, the supposed mechanism driving the diclofenac release is the matrix diffusion-controlled release, with the polymeric CGP-PPG nanoparticles acting as a controlled delivery device for diclofenac [21].

## 4. Limitations and Future Perspectives

Despite the good results for diclofenac encapsulation, tests regarding the efficiency of loading for other NSAIDs molecules with different hydrophilicity/hydrophobicity are required to confirm the potential of the CGP-PPG nanoparticles as drug carriers. Future tests might be conducted to evaluate the use of CGP-PPG@diclofenac via transdermal administration or topical application, which can increase the application possibilities of this drug delivery system. In addition, ex vivo permeation studies and the evaluation of the release profile of the CGP-PPG@diclofenac nanoparticles in the plasma conditions can improve the information about the performance of drug release of these particles in the systemic circulation. Finally, comparative studies with a commercial formulation and in vivo tests can be carried out to clarify possible modifications in the pharmacokinetic and pharmacodynamics parameters of the prepared nanoparticle.

## 5. Conclusions

In this study, the optimal preparation conditions required to obtain nanoparticles were optimized using the CCRD-RSM by fitting a quadratic model to the response data. Results evidenced that the increase in the speed of homogenization and the interaction between the polymers CGP and PPG contributed to produce nanoparticles with a lower size and higher loading of diclofenac. Using the optimized proportion of polymers and homogenization speed, it was possible to produce CGP-PPG@diclofenac showing an EE of 95.6%, with a hydrodynamic diameter of 275 nm, ζ-potential of −5.98 mV, and a particle average size of 321 nm. The diclofenac encapsulation was confirmed by FTIR and XRD analysis. The release of diclofenac sodium took place of a period of 68 h, following a typically anomalous transport mechanism. Therefore, the present study showed that nanoparticles obtained from cashew gum polysaccharide grafted with polypropylene glycol are promising materials for loading and drug release for molecules with a structure similar to diclofenac sodium, contributing to avoid the “peak and valley” problems associated with the oral therapy of these drugs.

## Figures and Tables

**Table 1:** Coded and actual levels of the variables used in the 2^3^ central composite rotatable design (CCRD) to produce the CGP-PPG@diclofenac nanoparticles.

Test n°	Coded Level of Variables			Actual Level of Variables		
	CGP ^1^	PPG	Speed	CGP (mg)	PPG (μL)	Speed (rpm)
1	−1	−1	−1	5.0	5.0	14,000
2	−1	−1	+1	5.0	5.0	22,000
3	−1	0	0	5.0	7.5	18,000
4	−1	+1	−1	5.0	10.0	14,000
5	−1	+1	+1	5.0	10.0	22,000
6	0	−1	0	10.0	5.0	18,000
7	0	0	−1	10.0	7.5	14,000
8 (c)	0	0	0	10.0	7.5	18,000
9	0	0	+1	10.0	7.5	22,000
10	0	+1	0	10.0	10.0	18,000
11	+1	−1	−1	15.0	5.0	14,000
12	+1	−1	+1	15.0	5.0	22,000
13	+1	0	0	15.0	7.5	18,000
14	+1	+1	−1	15.0	10.0	14,000
15	+1	+1	+1	15.0	10.0	22,000
16 (c)	0	0	0	10.0	7.5	18,000

^1^ CGP: cashew gum polysaccharide; PPG: polypropylene glycol; (c): central point.

**Table 2:** Results obtained in the 2^3^ CCRD for hydrodynamic diameter, polydispersity index, zeta potential, and drug entrapment efficiency for the CGP-PPG@diclofenac nanoparticles.

Test No.	Hydrodynamic Diameter (nm)	Polydispersity Index (PDI)	Zeta Potential (mV)	Encapsulation Efficiency (%)
1	896	0.51	−17.33	93.5
2	1209	0.52	−8.31	87.9
3	942	0.65	−2.99	78.3
4	4508	1.00	−11.77	77.6
5	660	0.52	−10.05	78.2
6	3170	0.93	−9.30	96.6
7	2097	0.48	−8.88	91.7
8 (c)	696	0.62	−5.32	90.6
9	544	0.43	−8.47	91.6
10	635	0.46	−25.03	90.5
11	10080	0.95	−0.08	92.4
12	1936	0.71	−2.18	91.2
13	2708	0.63	−3.17	89.8
14	10000	1.00	−1.55	90.3
15	1052	0.62	−2.05	89.9
16 (c)	642	0.51	−17.33	90.5

Results were expressed as a mean of six determinations.

**Table 3:** Kinetic model data for the evaluation of diclofenac release from nanoparticles.

Kinetic Model	r^2^	AIC ^1^
Zero-order	0.9634	0.265
First order	0.8335	0.485
Higuchi	0.6963	0.393
Korsmeyer–Peppas	0.9975	0.234

^1^ AIC: Akaike information criterion (*n* = 6 determinations).

## Data Availability

The data presented in this study are available on request from the corresponding author.
